# Direct Connection to the ECMO Circuit versus a Hemodialysis Catheter Is Associated with Improved Urea Nitrogen Ultrafiltration during Continuous Renal Replacement Therapy for Patients on Extracorporeal Membrane Oxygenation

**DOI:** 10.3390/jcm12041488

**Published:** 2023-02-13

**Authors:** Anna L. Ciullo, Richard Knecht, Nicholas M. Levin, Nathan Mitchell, Joseph E. Tonna

**Affiliations:** 1 Division of Cardiothoracic Surgery, Department of Surgery, University of Utah Health, Salt Lake City, UT 84132, USA; 2 Department of Emergency Medicine, University of Utah Health, Salt Lake City, UT 84132, USA; 3 Department of Emergency Medicine, University of California Los Angeles, Los Angeles, CA 90095, USA; 4 Department of Medicine, Sanford University, Palo Alto, CA 94305, USA

**Keywords:** dialysis, CRRT, HD, ultrafiltration, renal failure

## Abstract

For patients on extracorporeal membrane oxygenation (ECMO) who require renal replacement therapy (RRT), dialysis can be achieved through a dedicated hemodialysis (HD) catheter or direct connection to the ECMO circuit. The relative effect of each on filtration efficacy is not known. We conducted a retrospective single-center analysis of patients on ECMO who required CRRT. We examined the outcomes of blood biomarkers and transmembrane filter pressures, comparing sessions by attachment approach. All analyses were clustered by patient. Of the 33 patients (7 ECMO access and 23 HD catheter access) that met the inclusion criteria, there were a total of 493 CRRT sessions (93 ECMO access and 400 HD catheter access). At the end of the first 12 h of CRRT therapy, the ECMO group had a greater rate of decline in serum BUN than the HD catheter access group (2.5 mg/dl (SD 11) vs. 2 mg/dl (SD 6), *p* = 0.035). Additionally, the platelet level was significantly higher in the ECMO group compared to the HD catheter access group after 72 h (94.5 k/uL (SD 41) vs. 71 k/uL (SD 29), *p* = 0.008). Utilizing the ECMO circuit as direct venous access for CRRT was associated with some improved filtration proximal outcomes.

## 1. Introduction

Critically ill patients requiring continuous renal replacement therapy (CRRT) are at increased risk of both mechanical and infectious complications from the placement and maintenance of vascular access catheters, as well as from CRRT circuit changes, respectively [1,2]. While 36% of critically ill patients will require CRRT during their intensive care unit (ICU) stay, patients also requiring extracorporeal membrane oxygenation (ECMO) are at further increased risk of acute kidney injury (AKI) and thus the mechanical and infectious complications associated with initiating and supporting CRRT [3,4,5].

Vascular access for CRRT is traditionally accomplished through the placement of an additional, temporary dedicated central-venous catheter. These large-bore catheters have been associated with subsequent vascular stenosis, which increases the risk of eventual loss of additional access points in these patients [6]. As patients requiring CRRT are subsequently at increased risk of long-term hemodialysis, strategies to decrease vascular access attempts and stenosis are desirable [7].

Patients receiving ECMO therapy provide a potential opportunity to avoid dedicated central venous-access for the purposes of dialysis alone by connecting the CRRT circuit directly to the ECMO circuit ports [8,9,10,11]. Multiple studies have described the theory, physical interface and practical considerations of connecting the CRRT circuit to the ECMO circuit, and one study examined the duration of circuit life between this approach and a traditionally accessed central venous catheter. To our knowledge, no studies have examined the ultrafiltration efficacy associated with each approach. As the prevalence of patients who undergo ECMO each year increases, of whom 50% will require CRRT, if the filtration efficacy of direct ECMO connection is comparable to using a dedicated HD catheter, use of this approach could obviate the additional cost and procedural complications of placing and using a dedicated HD catheter for these patients [5,12].

To inform the decision on the optimal CRRT connection in patients requiring both ECMO and CRRT, we analyzed the outcomes of filtration efficacy using a large number of CRRT filter sessions from a single academic medical center.

## 2. Materials and Methods

### 2.1. Study Design

This was an observational, retrospective cohort study at a single tertiary academic medical center. We screened all patients admitted to the cardiovascular intensive care unit (CVICU) from 1 May 2016 through 10 September 2017 to identify adult patients (age ≥ 18 years) requiring CRRT and ECMO simultaneously. Patients were enrolled retrospectively after Institutional Review Board approval with waiver of consent. There were no exclusion criteria.

### 2.2. Covariates and Outcomes

The primary outcome was the change in hematologic markers during CRRT. These markers were chosen as a proxy for ultrafiltration efficacy. We assessed the change in variables from the beginning of each filter session until the end of each session.

### 2.3. Statistical Analysis

Descriptive statistics, including the mean (SD) and median (interquartile range), were used to assess patient characteristics. Categorical characteristics were compared using the chi-square test or Fisher exact test. Continuous characteristics were compared using an independent samples *t*-test. For variables repeated within patients, to account for clustering, we utilized mixed effects regression, clustered by patient. Coefficients, 95% CIs and *p*-values were reported for all models. Statistical analyses were conducted in STATA v15 (College Park, TX, USA). Significance was assessed at the 0.05 level, and all tests were two-tailed.

## 3. Results

### 3.1. Patient Demographics

Of the 261 patients screened, we identified 33 patients (7 ECMO access and 23 HD catheter access) that met inclusion criteria from 3 May 2016 until 9 September 2017, resulting in 493 CRRT sessions (93 ECMO access and 400 HD catheter access). Patient characteristics are listed in Table 1. Briefly, patients were 57 years old (IQR 50–64) and 12 (36.6%) were female and 21 (63.6%) were males. The overall mean SOFA score at the start of CRRT was 12 (IQR 10–14), with an average length of stay of 22 days (IQR 15–43). There was no statistically significant difference in all baseline characteristics or ICU variables between the ECMO access and HD catheter access groups.

### 3.2. Differences in Serum Creatinine

The median serum creatinine at the termination of all CRRT sessions was 1.58 mg/dL (IQR 1.22–2.15). At the termination of CRRT sessions, the ECMO access group had a median serum creatinine of 1.31 mg/dL (IQR 0.92–1.75), while the HD catheter access group had a median serum creatinine of 1.65 (IQR 1.26–2.19) (*p* = 0.381) (Table 2).

### 3.3. Changes in Serum Blood Urea Nitrogen (BUN)

**Figure 1:**
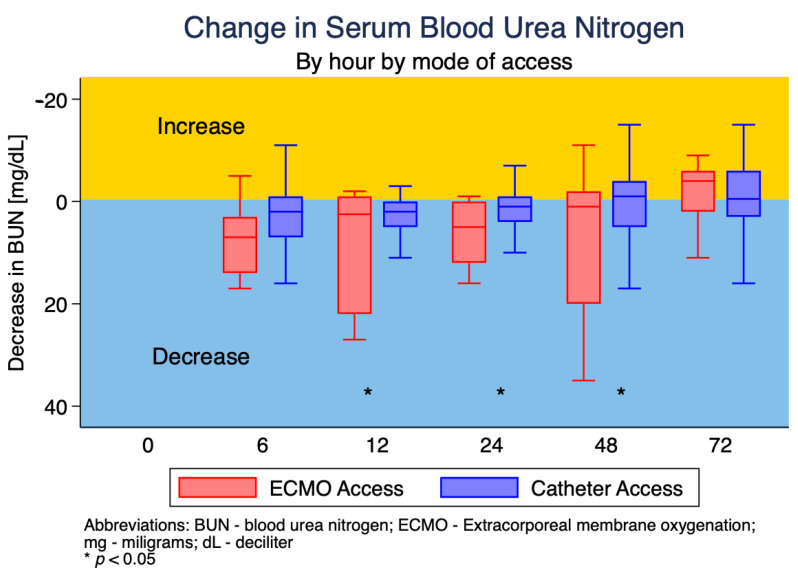
Change in serum BUN each hour according to connection type.

Serum BUN was found to decrease more rapidly in the ECMO access group vs. the HD catheter access group at the time points of 12, 24 and 48 h (*p* < 0.05). The change in serum BUN over time is graphically represented in Figure 1.

### 3.4. Differences in Platelet Count

**Figure 2:**
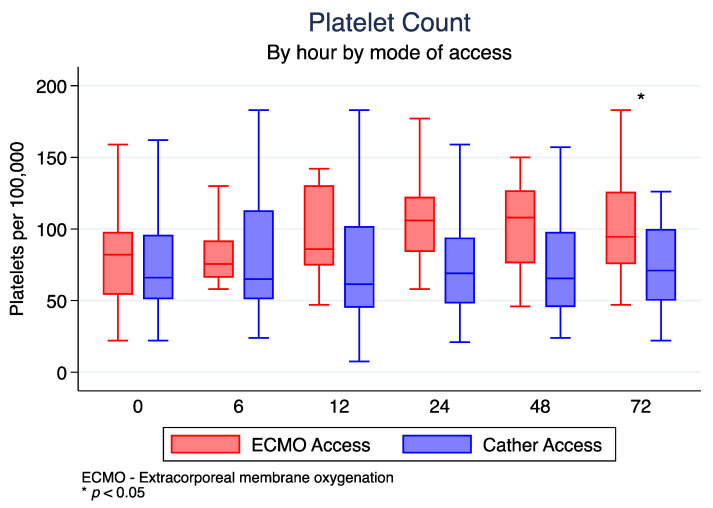
Blood platelet level each hour according to connection type.

The ECMO access group had a higher median platelet count at the end of all CRRT runs compared to the HD catheter access group (89 k/uL (IQR 73–113) vs. 66 k/uL (IQR 49–99); *p* < 0.01) (Table 2). This was most reflected at 72 h, where the ECMO access group was significantly different from the HD catheter group (*p* = 0.008) (Table 3). The platelet level at each hour according to connection type is represented in Figure 2.

### 3.5. Differences in Transmembrane Pressure

**Figure 3:**
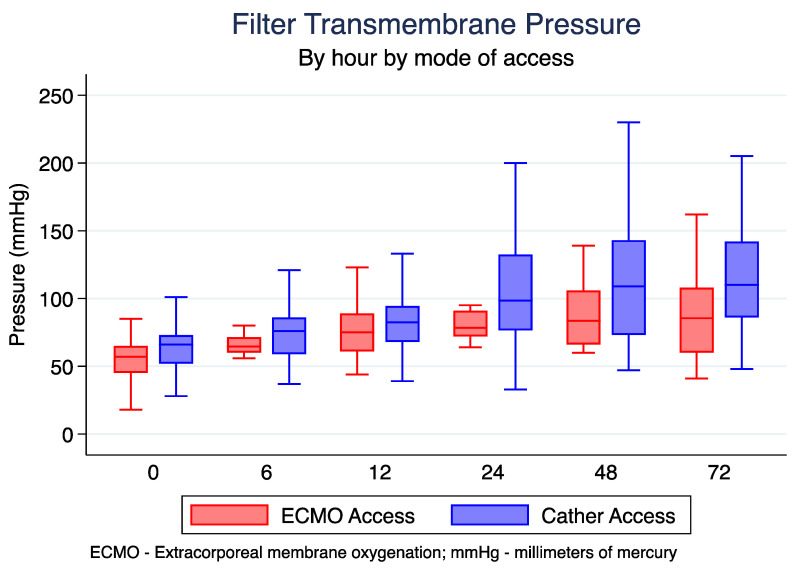
Filter transmembrane pressure each hour according to connection type.

CRRT filter transmembrane pressure (TMP) was lower at the termination of CRRT runs in the ECMO access group vs. the HD catheter access group (70 mmHg (IQR 60–86) vs. 80 mmHg (IQR 65–106), *p* = 0.215) (Table 2). Overall, TMP was lower across the duration of CRRT therapy at all time intervals, but this did not reach statistical significance (Figure 3).

## 4. Discussion

Utilizing the ECMO circuit as direct venous access for patients concurrently undergoing CRRT and ECMO therapy at our large referral ECMO center was associated with some improved filtration outcomes, such as improved BUN levels and higher platelet counts, when CRRT was administered via the ECMO circuit as opposed to a separate vascular access site. Additionally, we observed improved serum creatinine levels and lower CRRT filter transmembrane pressures, but these variables did not reach statistical significance.

There are several advantages to using the ECMO circuit as circulatory access for the CRRT filter without the need for an additional intravenous catheter. These advantages include: less systemic anticoagulation, decreased access sites that place the patient at risk of infection, decreased time to CRRT initiation and decreased procedural risks associated with a patient on ECMO [11,13]. Other potential advantages of using the ECMO circuit for vascular access and its direct effects on the CRRT machine and inline filter are not well understood. Additionally, we have described counts of clopidogrel, aspirin, anticoagulant type and access site in the Supplementary Materials, split by CRRT access point (Tables S1–S4 (Supplementary Materials)).

Compared to a study by de Tymowski et al., we did observe some improved ultrafiltration proximal outcomes among our patients receiving CRRT via their ECMO circuit [14]. Serum BUN was lower at the end of CRRT sessions for the ECMO group, and the platelet count was higher. This suggests that ECMO circulatory access creates an environment where waste products are removed more efficiently while maintaining stable platelet counts. We speculate that there was less platelet consumption in the ECMO group as a result of less resistance, given the difference in catheter radius between that of the hemodialysis and ECMO catheters. We also acknowledge that the difference in serum BUN between groups may be due to unmeasured confounders, including urea generation or precursor supply, rather than ultrafiltration efficacy. Examining the multivariate analysis, PTT was also lower in the ECMO after 24 h. Further studies would need to confirm these findings, but again, we suggest that the ECMO group displayed favorable conditions that enhanced CRRT function while preserving platelet function and a lower average aPTT.

There are several limitations to our study. As the study is retrospective and non-randomized, we cannot comment on causation and potential confounders that may exist among the groups. Additionally, given our small sample size, our data are at risk of a type II error. There may be a greater difference between the groups, that was not captured due to our small sample size.

The use of ECMO has been increasing in the United States over the last decade and has now become a prevalent mode of life-sustaining therapy for critically ill patients with multiple diseases [7]. A high proportion of patients undergoing ECMO therapy will also acquire an AKI during their treatment, necessitating CRRT [8]. Therefore, a simple, safe and effective method of obtaining circulatory access for inline CRRT is warranted.

## 5. Conclusions

Our results demonstrate that the direct connection of the CRRT circuit to the ECMO circuit was associated with improved filtration efficacy for select biomarkers. A further prospective study is warranted to confirm or refute our findings and to examine the effect on filter life.

## Figures and Tables

**Table 1:** Patient Characteristics.

Variable ^1^	All Admissions (n = 33)	ECMO Access (n = 7)	HD Catheter Access (n = 26)	*p*-Value
**Baseline Variables**				
Age, mean (IQR)	57 (50–64)	54 (33–61)	57.5 (50–65)	0.338
Female, n (%)	12 (36.4%)	4 (57.14%)	8 (30.77%)	0.198
Male, n (%)	21 (63.6%)	3 (42.86%)	18 (69.23%)	
**Characteristics at the start of CRRT**				
SOFA score	12 (10, 14)	13 (7, 15)	12 (10, 14)	0.695
Serum creatinine (mg/dL)	2.53 (2.09, 327)	1.91 (1.18, 3.38)	2.58 (2.29, 3.27)	0.385
Serum BUN (mg/dL)	52 (37, 76)	55 (39, 959)	50 (36, 83)	0.832
Hemoglobin (g/dL)	9.1 (8.1, 9.8)	8.2 (7.8, 8.3)	9.2 (8.2, 10)	0.109
Platelet count (k/uL)	69.5 (51, 96)	63 (25, 82)	72 (51, 122)	0.229
**ICU Variables**				
ICU LOS, days	22 (15, 43)	21 (14, 29)	22 (15, 51)	0.695
Died in ICU, n (%)	21 (63.64%)	4 (66.67%)	17 (62.96%)	1.0

Abbreviations: SD = standard deviation; ICU = intensive care unit; IQR = interquartile range; LOS = length of stay. ^1^ median (IQR) unless specified.

**Table 2:** Patient Blood and Filter Characteristics by Location of CRRT Access.

Variable ^1^	All Admissions (n = 33)	ECMO Access (n = 7)	HD Catheter Access (n = 26)	*p*-Value ^2^
**Laboratory values at end of each session**				
Serum creatinine (mg/dL)	1.58 (1.22, 2.15)	1.31 (0.92, 1.75)	1.65 (1.26, 2.19)	0.381
Serum BUN (mg/dL)	43 (34, 57)	43 (33, 54)	44 (34, 58)	0.879
Hemoglobin (g/dL)	8.9 (8.3, 9.4)	8.7 (8.05, 9.55)	8.9 (8.4, 9.4)	0.437
Platelet count (1000/microliter)	71 (51, 104)	89 (73, 113)	66 (49, 99)	0.257
TMP (mmHg)	78 (63, 101)	70 (60, 86.5)	80 (65, 106)	0.215
Inflow pressure (mmHg)	−42 (−57, −27)	185 (153–213)	−47 (−60, −36)	<0.001
Outflow pressure (mmHg)	51 (33–64)	−66 (−81, −48)	55 (44, 67)	<0.001
Decrease in serum BUN over 6 h (mg/dL)	1 (−2, 5)	2.5 (−1, 12.5)	1 (−2, 5)	0.054
Decrease in serum creatinine over 6 h (mg/dL)	0.07 (0, 0.23)	0.07 (0, 3)	0.07 (0, 0.22)	0.137
Average aPTT (seconds)	50 (41, 69.5)	49 (42, 75)	50 (40, 67)	0.456
Average platelet count (1000/microliter)	71 (51, 104)	89 (73, 113)	66 (49, 99)	0.257
Average hemoglobin (g/dL)	8.9 (8.3, 9.4)	8.7 (8.05, 9.55)	8.9 (8.4, 9.4)	0.437
ECMO Flow during Session (L)	4.45 (3.73, 4.98)	4.28 (3.85, 4.54)	4.5 (3.7, 5.05)	0.430

Abbreviations: SD = standard deviation; ICU = intensive care unit; IQR = interquartile range; LOS = length of stay; TMP = transmembrane pressure; SCr = serum creatinine; BUN = serum blood urea nitrogen. ^1^ median (IQR) unless specified; ^2^ *p*-Value from univariate mixed effects model, clustered by the patient.

**Table 3:** Serum Biomarker Values Over Time.

Variable ^1^	All Admissions (n = 33)	ECMO Access (n = 7)	HD Catheter Access (n = 26)	*p*-Value
**Average SCr (mg/dL)**				
Hour 0	1.91 (1.32, 2.5)	1.63 (1.19, 2.34)	2 (1.46, 2.41)	0.801
Hour 6	1.98 (1.35, 2.41)	1.63 (1.19, 2.34)	2.00 (1.46, 2.41)	0.497
Hour 12	1.54 (1.22, 2.06)	1.31 (0.90, 1.73)	1.65 (1.25, 2.08)	0.349
Hour 24	1.63 (1.22, 2.06)	1.06 (0.86, 1.36)	1.68 (1.27, 2.13)	0.267
Hour 48	1.32 (1.05, 1.79)	1.02 (0.91, 1.24)	1.49 (1.19, 1.93)	0.260
Hour 72	1.23 (0.97, 1.60)	1.00 (0.93, 1.42)	1.29 (1.07, 1.63)	0.556
**Average BUN (mg/dL)**				
Hour 0	46 (37, 62)	48 (31.5, 62)	50 (36, 62)	0.490
Hour 6	49 (36, 62)	48 (31.5, 62)	50 (36, 62)	0.801
Hour 12	42 (34, 55)	45 (33, 60)	42 (34, 55)	0.765
Hour 24	42 (33, 54)	41 (31, 50)	42.5 (34, 57)	0.685
Hour 48	43 (30, 50)	34 (29, 43)	45 (30, 52)	0.458
Hour 72	41 (33, 48)	39 (31.5, 45)	42 (33, 51)	0.459
**Average TMP (mmHg)**				
Hour 0	64.5 (50.5, 72)	64.5 (60, 71.5)	76 (59, 86)	0.154
Hour 6	72.5 (60, 84)	64.5 (60, 71.5)	76 (59, 86)	0.248
Hour 12	81 (67, 93)	75 (61, 89)	82.5 (68, 94.5)	0.451
Hour 24	91.5 (73.5, 125)	78.5 (72, 91)	98.5 (76.5, 132.5)	0.416
Hour 48	106 (71, 141)	83.5 (66, 106)	109 (73, 143)	0185
Hour 72	101 (70, 136)	85.5 (60, 108)	110 (86, 142)	0.193
**Average PTT (seconds)**				
Hour 0	49.5 (39.5, 69.5)	54.4 (44.5, 69)	47 (38, 63)	0.344
Hour 6	47 (40, 63)	54.5 (44.5, 69)	47 (38, 63)	0.476
Hour 12	50 (41,70)	61.5 (44, 79)	50 (41, 66)	0.625
Hour 24	49 (40, 70)	46 (39, 73)	50 (40, 70)	0.309
Hour 48	53 (41, 69)	48.5 (41, 79)	54 (42, 66)	0.908
Hour 72	54 (42, 73)	49 (42.5, 75)	57.5 (42, 72)	0.293
**Average PLT (k/uL)**				
Hour 0	69.5 (51, 96)	75.5 (66, 92)	65 (51, 113)	0.152
Hour 6	67 (54, 102)	75.5 (66, 92)	65 (51, 113)	0.876
Hour 12	67 (49, 107.5)	86 (74.5, 130.5)	61.5 (45, 102)	0.819
Hour 24	77 (51, 108)	106 (84, 122.5)	69 (48, 94)	0.985
Hour 48	69.5 (48, 111)	108 (76, 127)	65.5 (45.5, 98)	0.734
Hour 72	78 (52, 102)	94.5 (75.5, 126)	71 (50, 100)	0.008
**Change in SCr (mg/dL)**				
Hour 6	0.18 (0, 0.38)	0.27 (0, 0.48)	0.17 (0, 0.36)	0.318
Hour 12	0.11 (0.04, 0.22)	0.08 (0.02, 0.62)	0.12 (0.05, 0.22)	0.069
Hour 24	0.045 (0.005, 0.14)	0.06 (0.03, 0.25)	0.04 (−0.03, 0.14)	0.171
Hour 48	0.05 (−0.03, 0.22)	0.045 (−0.035, 0.335)	0.05 (−0.02, 0.22)	0.53
Hour 72	0.01 (−0.06, 0.18)	−0.01 (−0.17, 0.07)	0.025 (−0.035, 0.215)	0.167
**Change in BUN (mg/dL)**				
Hour 6	3 (−1, 8)	7 (3, 14)	2 (−1, 7)	0.360
Hour 12	2 (0, 5)	2.5 (−1, 22)	2 (0, 5)	0.035
Hour 24	1.5 (−1, 5)	5 (0, 12)	1 (−1, 4)	0.039
Hour 48	0 (−3, 6)	1 (−2, 20)	−1 (−4, 5)	0.009
Hour 72	−1 (−6, 3)	−4 (−6, 2)	−0.5 (−6, 3)	0.744

Abbreviations: SD = standard deviation; SD = standard deviation; ICU = intensive care unit; IQR = interquartile range; LOS = length of stay; TMP = transmembrane pressure; SCr = serum creatinine, BUN = serum blood urea nitrogen; ^1^ median (IQR) unless specified.

## Data Availability

Data and code are available from the authors upon reasonable request.

## Supplementary Materials

The following supporting information can be downloaded at: https://www.mdpi.com/article/10.3390/jcm12041488/s1, Figure S1: Picture of CRRT access points on the ECMO circuit; Table S1: Descriptive summary of clopiogrel (Plavix) use by access approach; Table S2: Descriptive summary of aspirin use by access approach; Table S3: Descriptive summary of anticoagulant use by access approach; Table S4: Descriptive summary of access site for catheter access patients.
