# Supplementary material for: Direct Connection to the ECMO Circuit versus a Hemodialysis Catheter Is Associated with Improved Urea Nitrogen Ultrafiltration during Continuous Renal Replacement Therapy for Patients on Extracorporeal Membrane Oxygenation
Source: J Clin Med. 2023 Feb 13;12(4):1488. doi: 10.3390/jcm12041488 (PMC9964054; doi:10.3390/jcm12041488)
Supplement: Supplementary file 1 [file jcm-12-01488-s001.zip › jcm-2013158-supplementary.pdf]

Journal of
Clinical Medicine

MDPI

Supplementary

Direct Connection to the ECMO Circuit Versus a Hemodialysis Catheter Is Associated with Improved Urea Nitrogen Ultrafiltration during Continuous Renal Replacement Therapy for Patients on Extracorporeal Membrane Oxygenation

Anna L. Ciullo [1,2,†], Richard Knecht [3,†], Nicholas M. Levin [4], Nathan Mitchell [2] and Joseph E. Tonna [1,2,*]

[1] Division of Cardiothoracic Surgery, Department of Surgery, University of Utah Health, Salt Lake City, UT 84132, USA
[2] Department of Emergency Medicine, University of Utah Health, Salt Lake City, UT 84132, USA
[3] Department of Emergency Medicine, University of California Los Angeles, Los Angeles, CA 90095, USA
[4] Department of Medicine, Sanford University, Palo Alto, CA 94305, USA
* Correspondence: joseph.tonna@hsc.utah.edu
† These authors contributed equally to this work.

Supplemental Results

Table S1: Descriptive summary of clopiogrel (Plavix) use by access approach
Table S2: Descriptive summary of aspirin use by access approach
Table S3: Descriptive summary of anticoagulant use by access approach
Table S4: Descriptive summary of access site for catheter access patients
Figure S1: Picture of CRRT access points on the ECMO circuit

Table S1. Descriptive summary of clopiogrel (Plavix) use by access approach.

Key
frequency<br>*column percentage*

CRRT Circuit Access Location	Plavix None	Plavix 75mg	Total
ECMO Access	7<br>24.14	0<br>0.00	7<br>21.21
Catheter Access	22<br>75.86	4<br>100.00	26<br>78.79
Total	29<br>100.00	4<br>100.00	33<br>100.00

Table S2. Descriptive summary of aspirin use by access approach.

Key
frequency<br>*column percentage*

CRRT Circuit Access Location	ASA None	ASA 81mg	Total
ECMO Access	3<br>25.00	4<br>19.05	7<br>21.21
Catheter Access	9<br>75.00	17<br>80.95	26<br>78.79
Total	12<br>100.00	21<br>100.00	33<br>100.00

Table S3. Descriptive summary of anticoagulant use by access approach.

CRRT Circuit Access Location	anticoagH Heparin	anticoagH Citrate	Total
ECMO Access	3<br>20.00	0<br>0.00	3<br>17.65
Catheter Access	12<br>80.00	2<br>100.00	14<br>82.35
Total	15<br>100.00	2<br>100.00	17<br>100.00

Table S4. Descriptive summary of access site for catheter access patients.

If HD cath; site and type	Freq.	Percent	Cum.
No Data	6	18.18	18.18
DL HD Cath Right Chest	1	3.03	21.21
DL HD Cath Left IJ	11	33.33	54.55
DL HD Cath Right IJ	8	24.24	78.79
DL HD Cath Left Femoral	1	3.03	81.82
DL HD Cath Right Femoral	1	3.03	84.85
DL HD Cath Left Subclavian	3	9.09	93.94
DL HD Cath Right Subclavian	1	3.03	96.97
None this CRRT run	1	3.03	100.00
Total	33	100.00	

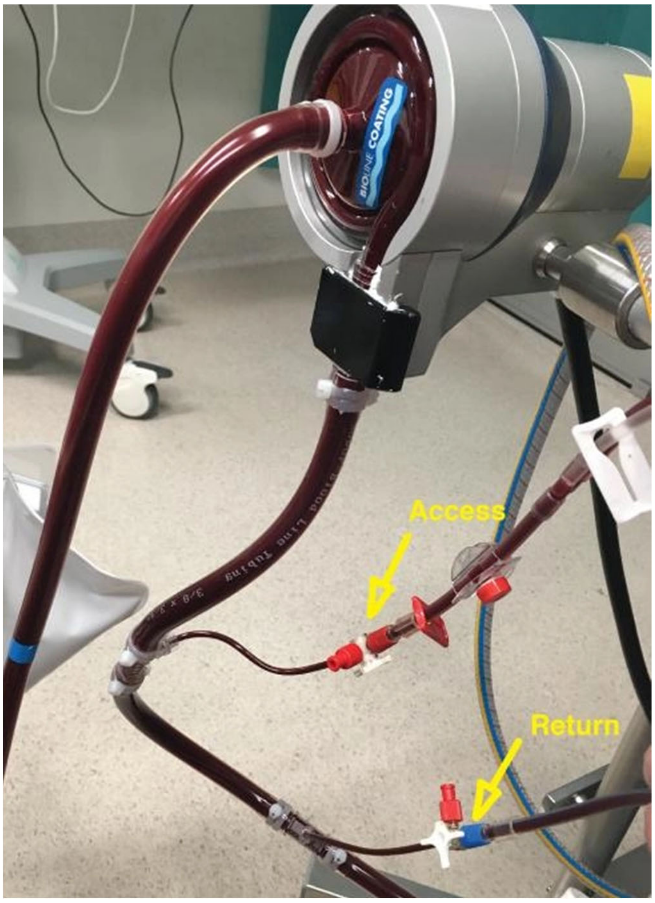

Figure S1. Picture of CRRT access points on the ECMO circuit.

The above image illustrates the two access points for CRRT on the ECMO circuit.
Image reference: https://ecmo.icu/daily-care-nursing-routine-crrt-and-plasmapheresis-connection/
